# Risk factors for incomplete resection with pharyngeal endoscopic submucosal dissection and long-term prognosis after resection

**DOI:** 10.1007/s00464-022-09820-8

**Published:** 2023-01-09

**Authors:** Yoshiki Sakaguchi, Yuki Saito, Mizuo Ando, Masafumi Yoshida, Osamu Fukuoka, Kenya Kobayashi, Dai Kubota, Daisuke Ohki, Hiroya Mizutani, Keiko Niimi, Yosuke Tsuji, Mitsuhiro Fujishiro, Tatsuya Yamasoba

**Affiliations:** 1 Department of Gastroenterology, Graduate School of Medicine, The University of Tokyo Hospital, Tokyo, Japan; 2 Department of Otolaryngology and Head and Neck Surgery, Graduate School of Medicine, The University of Tokyo Hospital, Tokyo, Japan

**Keywords:** Pharyngeal cancer, Endoscopic submucosal dissection, Incomplete resection, Prognosis, Diagnosis

## Abstract

**Background:**

Advances in endoscopic imaging technology have led to an increase in detection of superficial pharyngeal squamous carcinoma. Endoscopic submucosal dissection (ESD) has been reported to be effective for the treatment of these lesions, however there is still insufficient evidence on the long-term results of pharyngeal ESD.

**Methods:**

This is a single-center retrospective study of all cases of superficial pharyngeal cancer that underwent ESD as primary treatment between January 2010 and May 2022. A total of 83 lesions in 63 patients were analyzed.

**Results:**

The en bloc resection rate was 100%, and R0 resection rate was 59.0%, with an adverse event rate of 6.0%. During a mean observation period of 1134 days, there were 0 cases of disease-specific metastasis or death. However, the 5-year cumulative incidence of metachronous head and neck cancer after resection was 27.1% and the 5-year overall survival and 10-year overall survival after pharyngeal ESD were 87.0% and 69.6%, respectively. Of the 34 cases with non-R0 resection, local recurrence occurred in 8.8%. Location of lesion (*p* = 0.011), disparity between demarcation of the lesion with NBI and iodine staining (*p* = 0.026), and non-effective laryngeal elevation (*p* = 0.080) were risk factors for non-R0 resection.

**Conclusion:**

Pharyngeal ESD is effective and safe. Further studies are needed to improve and standardize indications and strategies for pharyngeal ESD.

Advances in endoscopic imaging technology and increasing attention to pharyngeal cancer during screening endoscopy have led to an increase in detection of superficial pharyngeal squamous carcinoma [1, 2]. This in turn has enabled minimally invasive methods of treatment for superficial pharyngeal cancer, such as endoscopic submucosal dissection (ESD) and endoscopic laryngo-pharyngeal surgery (ELPS) [3–11]. In these procedures, the horizontal demarcation of the lesions is diagnosed and marked using high-definition upper gastrointestinal (GI) endoscopes, and this is followed by local resection of the marked area. Thus, these methods are similar in concept, and have been reported to have comparable results. Especially ESD, which is widely performed worldwide for superficial neoplasms in the GI tract, has already been adopted in many facilities not only in Japan, but also in other countries in Asia and Europe [3–9].

As there is a low risk of recurrence even in cases where R0 resection cannot be pathologically confirmed [3, 12], there is limited literature assessing risk factors for the relatively low R0 resection rate in pharyngeal ESD. However, the risk of postoperative recurrence is not negligible in these cases, and there is a need to clarify risk factors for incomplete resection. Incomplete resection has been mainly attributed to cauterization of the edges of the specimen during the resection process [3, 6] but there is also a possibility that inaccurate extent diagnosis of superficial lesions may also attribute to the low R0 resection rate. While resection of wider margins may reduce the risk of incomplete resection, this may in turn lead to a deterioration of swallowing function. With an increasing number of facilities introducing pharyngeal ESD, albeit with a small number of patients [13], it is necessary to increase awareness of the efficacy and risks of pharyngeal ESD. In addition, it is necessary to optimize methods of diagnosis and treatment of superficial pharyngeal cancer. This study was performed with the aim to assess the efficacy and long-term prognosis of ESD for superficial pharyngeal cancer, and clarify risks associated with incomplete resection.

## Materials and methods

### Study design

This is a single‐center retrospective study of all consecutive cases of superficial pharyngeal cancer that underwent upper endoscopic resection at the University of Tokyo between January 2010 and May 2022. Informed consent for endoscopic treatment was obtained from all patients.

The study complied with the Declaration of Helsinki and was begun after approval by the Research Ethics Committee of the Graduate School of Medicine and Faculty of Medicine, The University of Tokyo. Informed consent was waived by the Research Ethics Committee of the Graduate School of Medicine and Faculty of Medicine, The University of Tokyo due to the retrospective design (#2487).

### Patients

All cases of endoscopic mucosal resection (EMR) and endoscopic submucosal dissection (ESD) performed between January 2010 and May 2022, were identified from the electronic medical records at the University of Tokyo Hospital. A total of 90 consecutive cases of endoscopic resection for superficial pharyngeal squamous cell carcinoma were extracted. As there were only four cases of EMR which were performed in 2010–2011, these cases were excluded from diagnosis. After further exclusion of 3 patients who had received salvage pharyngeal ESD for local recurrences after primary treatment of advanced pharyngeal cancer, a total of 83 lesions in 63 patients were included in the analysis.

### Treatment methods

#### Primary assessment and indications for ESD

All cases of pharyngeal cancer in this study underwent preoperative assessment with both a laryngoscope and high-vision upper GI endoscope (GIF-H260Z, GIF-290Z, GIF-H290I, GIF-1200 N; Olympus Co.). The visibility of the lesions with the laryngoscope, and accessibility with the GI endoscope were assessed. The horizontal extent of the lesions was assessed using the Valsalva method as required, and microvascular patterns of the lesions were also assessed with magnifying endoscopy when possible. The feasibility of pharyngeal ESD for each lesion were decided after careful discussion between the otolaryngologists and gastroenterologists. Pharyngeal cancers staged T4 or with lymph node metastases were considered contraindications for ESD.

### Preparation and assessment of horizontal extent

All ESD procedures were performed under general anesthesia by a team of otolaryngologists and expert gastroenterologists with experience of over 100 upper GI ESD. After induction of general anesthesia and intubation, a curved rigid laryngoscope (Nagashima Medical Instruments Company, Ltd, Tokyo, Japan) was inserted in all patients to provide a working space in the pharyngeal lumen. After complete visualization of the entire lesion was attained, detailed assessment of the target lesion was performed.

**Fig. 1:**
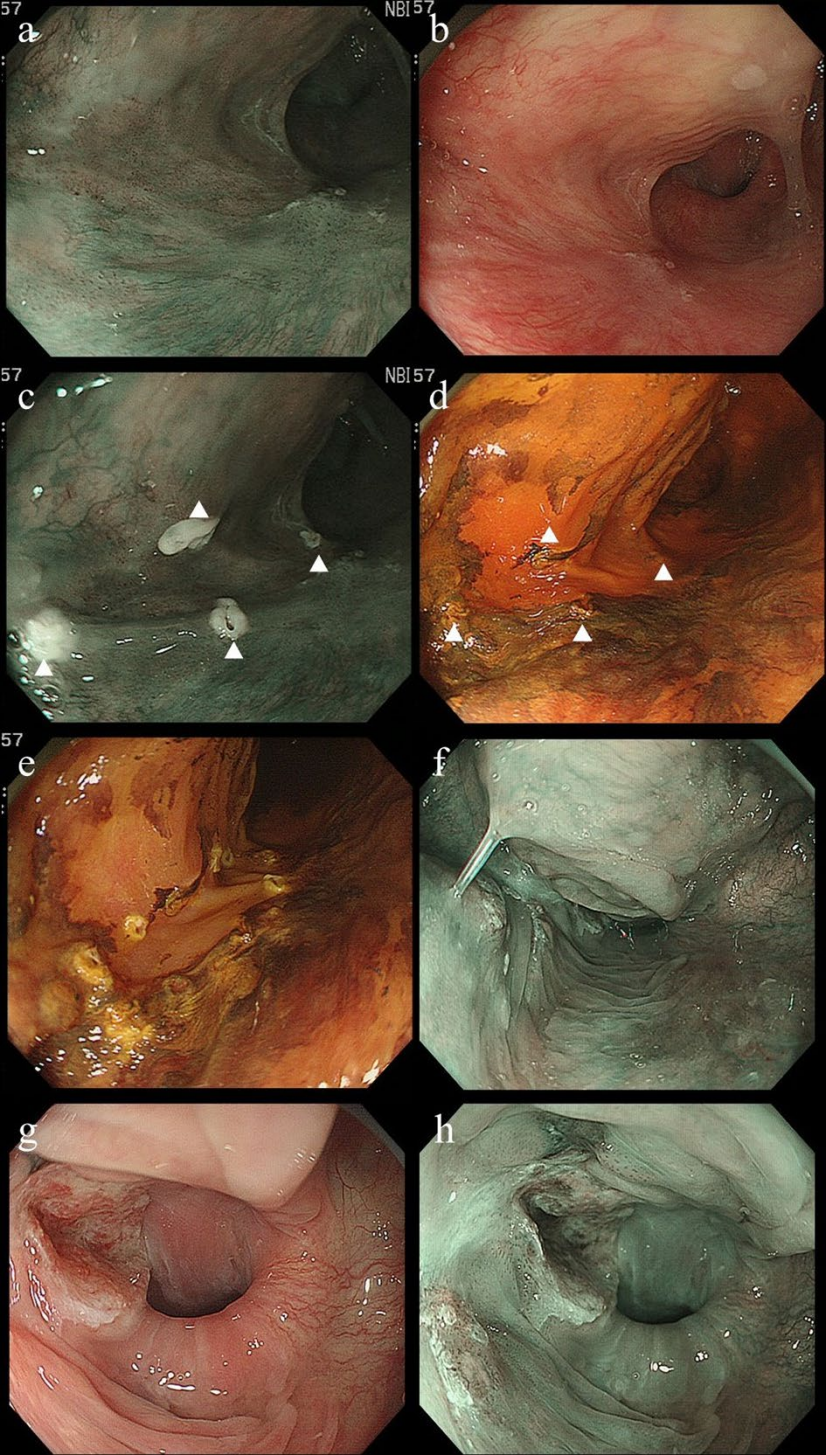
Local recurrence after pharyngeal ESD. **a** A subtle lesion in the left piriform sinus was detected, with clear demarcation by NBI, **b** although undetectable with white light observation. **c**) After marking of the perimeter of the lesion with NBI (marks are clarified by triangles), **d** Iodine staining was performed to confirm the horizontal extent, but non-stained areas extended far beyond the perimeter marks (marks are clarified by triangles). **e** After discussion between otolaryngologists and gastroenterologists, the horizontal extent visible by NBI was designated the target for resection, in order to minimize loss of pharyngeal function. **f** After careful follow-up, subtle local recurrence was detected 3 years later, and ESD was attempted. **g**, **h** However, multifocal recurrence with deep invasion adjacent to the esophageal orifice was detected after laryngeal elevation and total laryngectomy was required

Close observation of the targeted pharyngeal lesion(s) was performed using a magnifying upper GI endoscope (GIF-H260Z, GIF-290Z; Olympus Co.) with narrow-band imaging (NBI), followed by chromoendoscopy with iodine staining when possible. Iodine staining was abbreviated when the lesion was in close vicinity to the glottis, due to the risk of chemical pneumonitis in the event of iodine aspiration during intubation [14]. After demarcation of the horizontal extent of the lesion was fully assessed, the margin of the lesion was marked using a Dual Knife (KD-650L; Olympus Medical Systems Corp, Tokyo, Japan). In cases where the demarcation line of the lesion could not be diagnosed with high confidence due to an indiscernible demarcation line or a disparity between the demarcation line with NBI and iodine staining (Fig. 1), the target area for resection was determined after careful discussion between the otolaryngologist and gastroenterologist. In all cases, the target area for resection was determined with the objective of attaining R0 resection [15] while minimizing the risk of severe deterioration of swallowing function.

### ESD procedure

A single-channel upper GI endoscope (GIF-Q260J, GIF-H290T; Olympus Co.), a high-frequency generator VIO 300D or VIO-3 (ERBE Elektromedizin GmbH, Tübingen, Germany.), and the DualKnife (KD-655Q or KD-655L, Olympus Co.) were used for submucosal dissection. Submucosal injection of normal saline containing indigo carmine was used for each procedure. The use of hyaluronic acid was avoided in order to minimize edema of the pharynx and especially prevent long-lasting laryngeal edema, which can lead to prolonged intubation and/or otherwise unnecessary tracheostomy. Incision and dissection of the lesion was continued until completion of ESD.

In areas difficult to approach with the GI endoscope, counter-traction with laryngeal forceps was performed with the otolaryngologists’ cooperation in early years. Countertraction with hemoclip and dental thread was employed after 2016 to minimize damage to the specimen [16].

### Postoperative course

Oral diet was resumed at earliest postoperative day 1, after observation of the pharyngeal wound and movement with a laryngoscope. After beginning with a semi-solid diet, the patients were discharged after confirmation that solid foods could be swallowed without aspiration or severe pain.

### Histologic evaluation

Endoscopically resected specimens were formalin-fixed and sectioned at a 2–3 mm thickness. Tissue specimens were embedded in paraffin, sectioned at 4 μm, and stained with hematoxylin and eosin. Additional immunostaining with D2-40 was performed, as required. Tumor size, horizontal and vertical margins, and the presence of lymphovascular invasion were evaluated pathologically according to Japanese guidelines for head and neck cancer [15]. In cases with subepithelial invasion, tumor thickness was assessed as previously reported [6, 17].

### Follow-up after endoscopic resection

After pharyngeal ESD, all patients were followed up by both otolaryngologists to check for local and metachronous head and neck cancer, and also by gastroenterologists to screen for metachronous pharyngeal and esophageal cancer.

Follow-up with a laryngoscope every 3–6 months was performed for a minimum of 5 years after ESD. In addition, screening with an upper GI endoscope was also performed at 6-month intervals for as long as possible.

Incomplete resection, or lymphovascular invasion were considered indications for adjuvant therapy, and the options of adjuvant radiotherapy or chemoradiotherapy were discussed with the patient. In cases where the patient did not desire adjuvant therapy, follow-up was continued at close intervals, including diagnostic imaging with CT/MRI. Additional treatment was performed when definite signs of local recurrence or metastasis were detected.

### Clinical characteristics and definitions

Background factors, referral documents, endoscopic findings, and histopathological results were extracted from the medical records at the University of Tokyo Hospital. For patients with multiple sessions of pharyngeal ESD, background factors at time of discovery of the first pharyngeal lesion were assessed. Metachronous cancer rates and overall survival were assessed for all patients with the start of follow-up set at the date of the first pharyngeal ESD. Local recurrences were assessed for all lesions with the start of follow-up set at the date of each pharyngeal ESD.

### Statistical analyses

All continuous variables were compared using either the Student’s *t *test or Mann–Whitney *U *test, and categorical variables were compared using either the χ2 test or Fisher’s exact test as appropriate. Statistical significance was set at *p* value of < 0.05. All statistical analyses were performed using JMP Version 16.2 (SAS Institute Inc., Cary, NC, USA).

## Results

**Fig. 2:**
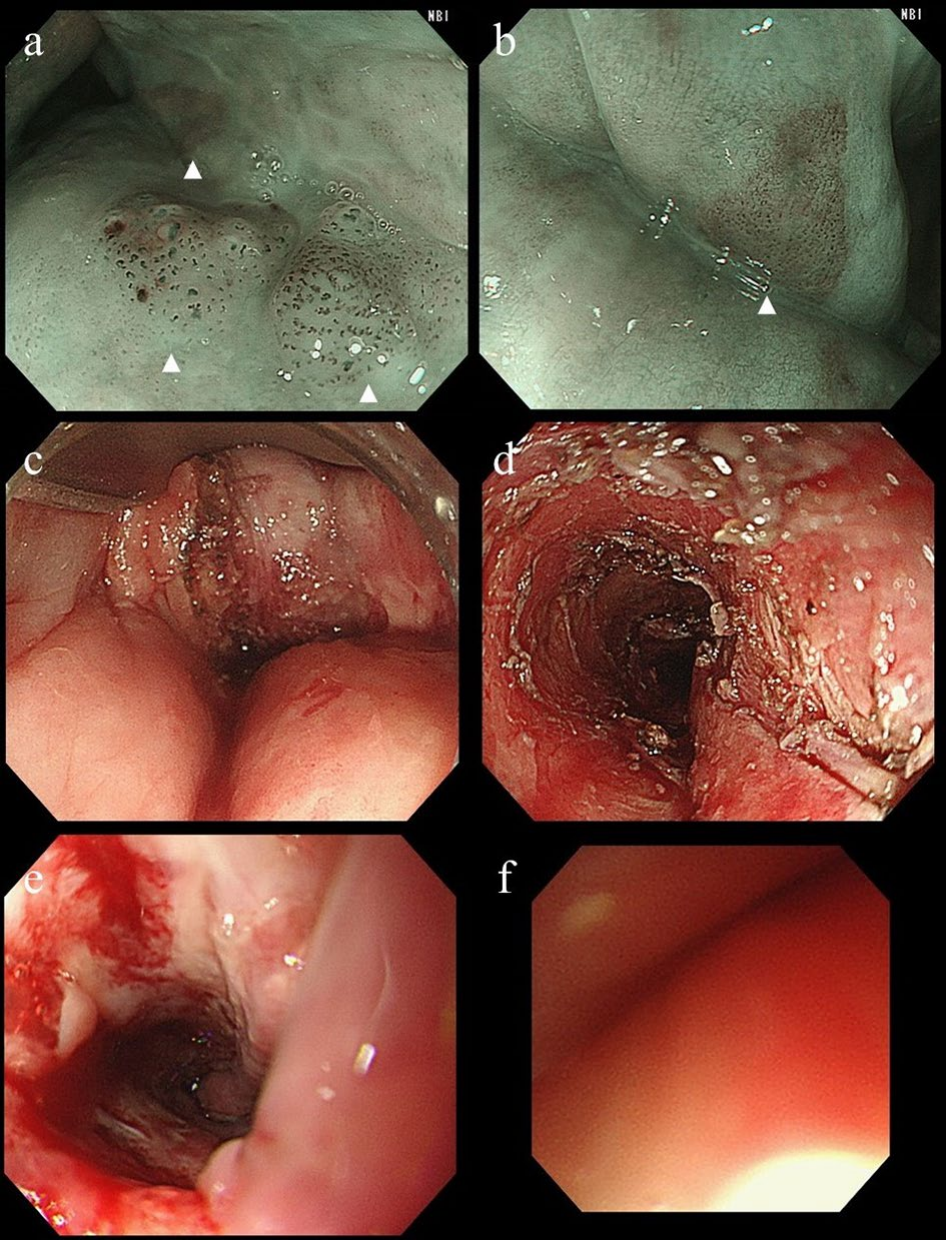
Severe stenosis after extensive ESD of the hypopharynx to esophagus. **a**, **b** Multiple squamous cell carcinomas were detected in the hypopharynx to the cervical esophagus. **c**, **d** Endoscopic submucosal dissection of these lesions was performed, resulting in resection of an extensive area from the hypopharynx to esophagus. **e** Upper GI endoscopy 1 month later demonstrated no stenosis, however the patient suffered from deterioration of swallowing function. **f** 6 months later, severe stenosis was found in the hypopharynx through which the endoscope could not pass

**Fig. 3:**
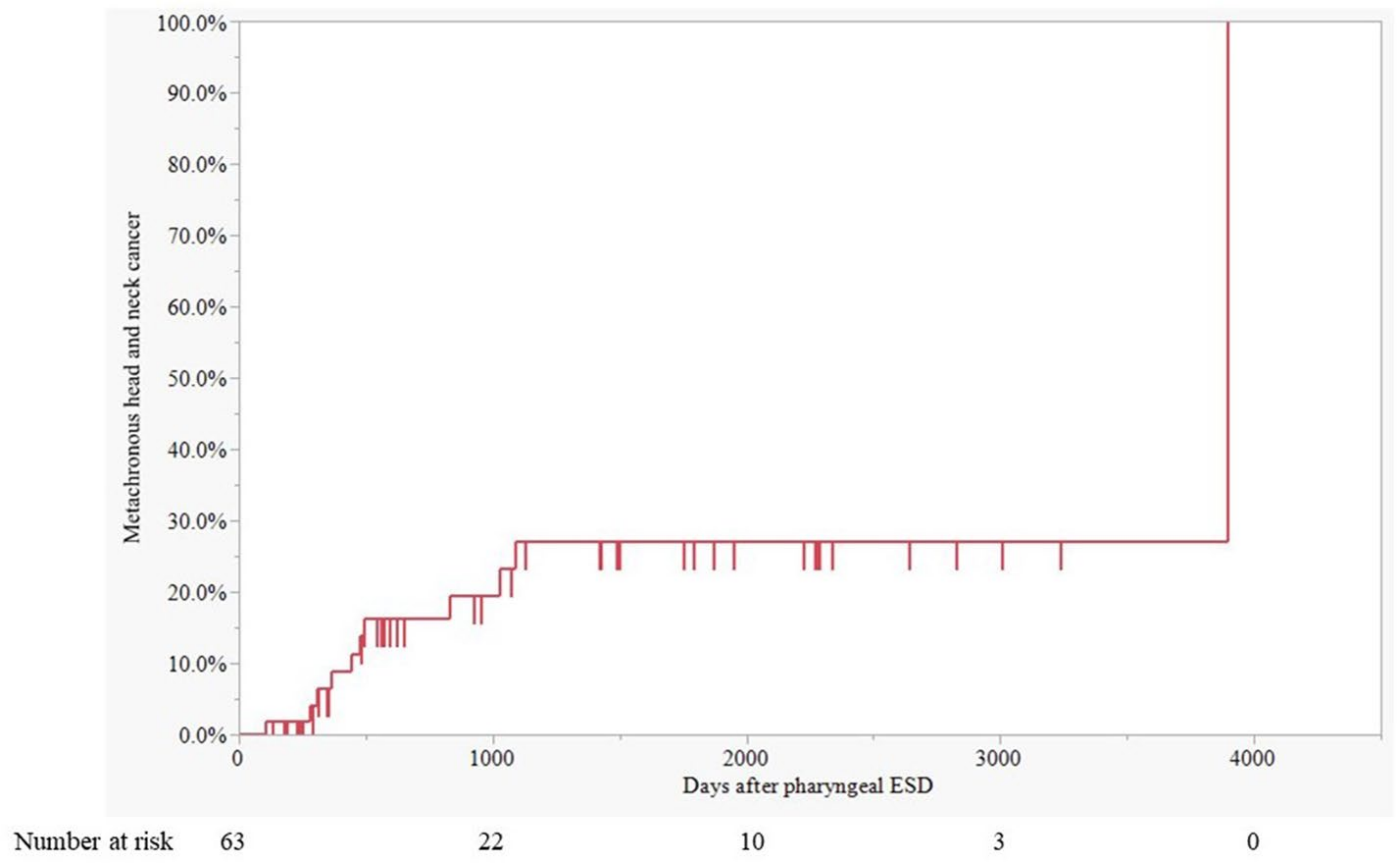
Cumulative metachronous head and neck cancer incidence after pharyngeal ESD

**Fig. 4:**
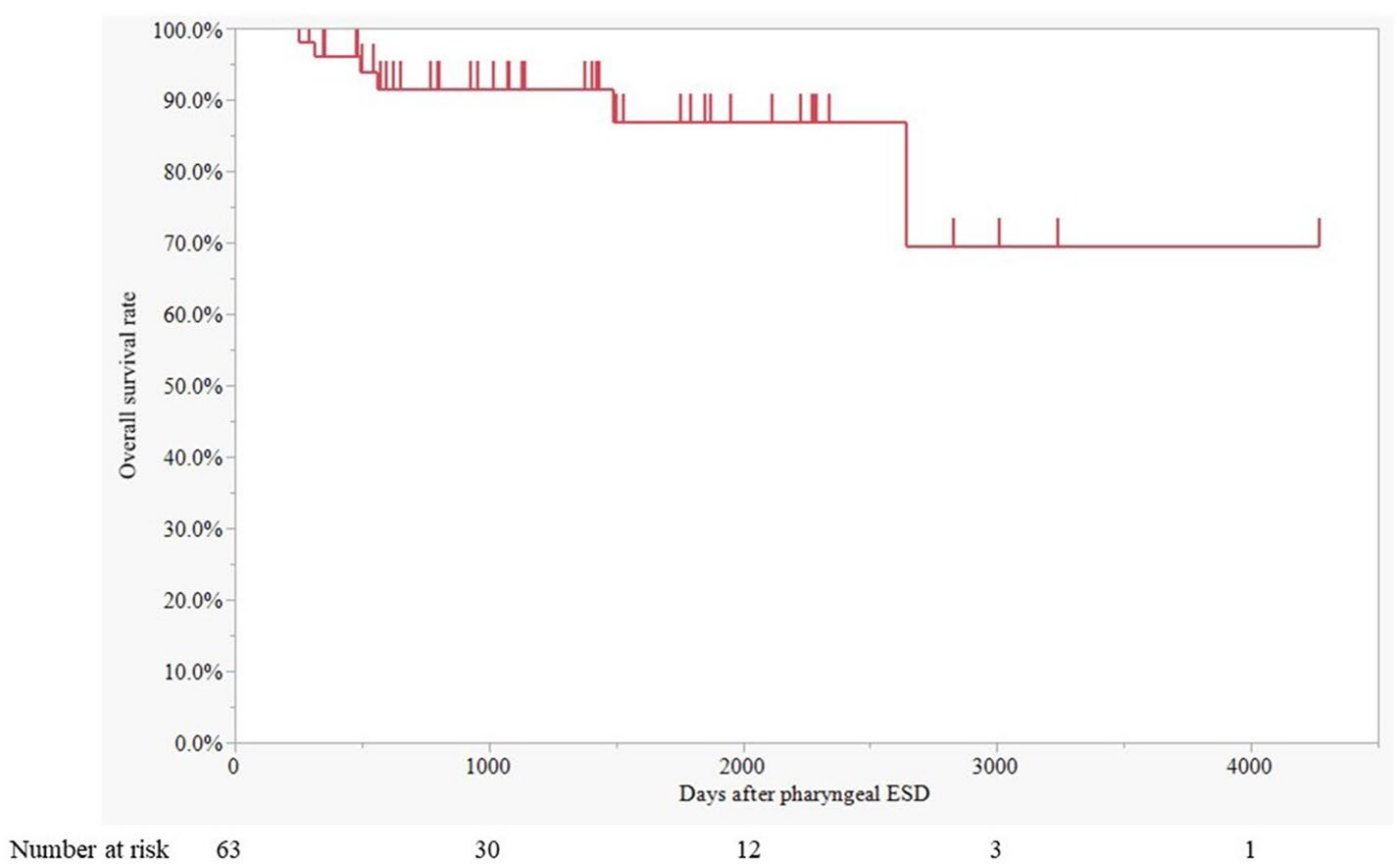
Overall survival after pharyngeal ESD

1. **Background characteristics**
  A total of 83 lesions in 63 consecutive patients were analyzed (Table 1). Concerning the background characteristics of the patients, 90.4% were male, with age at time of first pharyngeal ESD 68.0 ± 8.2 years old. The majority (85.7%) of the patients had a history of other cancer at the time when pharyngeal cancer was detected, with esophageal cancer being the most common (73.0%), followed by lung cancer (6.3%) and hepatic cancer (4.8%). The majority (79.4%) of the patients had both regular intake of alcohol and a history of smoking, with a high alcohol intake (63.7 ± 62.0 g/day), a high rate of flushers (60.4%), and a high rate of heavy smokers (23.2 ± 17.0 cigarettes/day, 30.5 ± 15.9 years). Only 4.8% of the patients did not have either regular alcohol intake or a history of smoking.
  Concerning the lesions, the majority (96.8%) were discovered during upper GI endoscopy, and the other 3.2% during laryngoscopy. Flat lesions were the most common macroscopic type (71.1%), and most lesions (79.5%) were located in the piriform sinus.
2. **Short-term efficacy and safety of pharyngeal ESD**
  The en bloc resection rate was 100%, and R0 resection rate was 59.0%. There were 3 (3.6%) cases with lymphovascular invasion after histologic evaluation of resected specimens. Adverse events occurred in 5 procedures (6.0%) with two cases of postoperative bleeding, one case of laryngeal edema requiring prolonged intubation, one case of facial iodine dermatitis after overflow of fluids from the oral cavity, and one case of severe deterioration of swallowing function after extensive resection. Both cases of postoperative bleeding were successfully controlled with hemostasis under general anesthesia. In the case with severe deterioration of swallowing function, dysphagia rehabilitation was not effective, and enteral nutrition through a gastrotomy was required until the patient eventually deceased due to aspiration pneumonia (Fig. 2).
3. **Prognosis and long-term results after resection**
  The follow-up period after ESD was 1134.8 ± 943.2 days.
  After non-curative pharyngeal ESD, only one case underwent adjuvant radiotherapy after R1 resection for a lesion with tumor thickness 3000 µm and lymphovascular invasion, resulting in no recurrence after 1951 days. All other cases with either R1 resection or lymphovascular invasion were followed up after careful discussion with the patient. Despite the low rate of patients who underwent adjuvant therapy, there were 0 cases of disease-specific metastasis or death.
  However, metachronous head and neck cancers occurred in a total of 11 cases (17.5%), and the 5-year metachronous head and neck cancer cumulative incidence rate was 27.1% (Fig. 3). The 5-year overall survival and 10-year overall survival were 87.0% and 69.6%, respectively (Fig. 4).
  Concerning local recurrences, of the 34 cases with non-R0 resection, local recurrence occurred in 3 cases (8.8%). Of these two lesions were managed by additional local resection, with no concurrent recurrences. However one case of local recurrence was not discovered until the remaining lesion had reached an advanced stage, and ultimately required total laryngectomy (Fig. 1). There were no cases of local recurrence after pathologically confirmed R0 resection.
4. **Risk factors associated with positive margins**
  As there is a risk of local recurrence after resection with positive margins, risk factors associated with positive margins were assessed. Location of lesion was a significant risk factor (*p* = 0.011), with higher R0 resection rates in relatively straight areas (posterior and lateral walls of the oropharynx and hypopharynx) in comparison to areas with complicated structures (piriform sinus and base of tongue) (Table 2). In addition, there was a higher R0 resection rate in cases where demarcation of the lesion was identical for NBI and iodine staining, compared to cases with disparity between these modalities (73.0% vs 45.2%, *p* = 0.025). In addition, there were higher R0 resection rates in cases where the entire lesion could be visualized after laryngeal elevation compared to cases where laryngeal elevation was not effective (58.3 vs 0%, *p* = 0.080).

**Table 1:** Background factors for pharyngeal ESD

Patient characteristics	*n* = 63
Age mean ± SD	68.0 ± 8.2
Gender male *n* (%)	57 (90.4%)
Method of detection	
Upper GI endoscope *n* (%)	61 (96.8%)
Laryngoscope *n* (%)	2 (3.2%)
History of cancer at time of detection	54 (85.7%)
Esophageal cancer *n* (%)	46 (73.0%)
Lung cancer *n* (%)	4 (6.3%)
Hepatic cancer *n* (%)	3 (4.8%)
Other cancer *n* (%)	6 (9.5%)
Social background	
Alcohol intake g/day, mean ± SD	63.7 ± 62.0
Flusher, yes *n* (%)^a^	26 (60.4%)
Current drinker, yes *n* (%)	47 (74.6%)
Smoking amount cigarettes/day, mean ± SD	23.2 ± 17.0
Smoking period years, mean ± SD	30.5 ± 15.9
Current smoker, yes *n* (%)	16 (26.2%)
Observation period after first pharyngeal ESD days, mean ± SD	1134.8 ± 943.2
Tumor characteristics	*n* = 83
Macroscopic type	
0-I *n* (%)	2 (2.4%)
0-IIa *n* (%)	22 (26.5%)
0-IIb *n* (%)	59 (71.1%)
Location *n* (%)	
Hypopharynx	
Piriform synus	66 (79.5%)
Posterior wall of hypopharynx	5 (6.0%)
Postcricoid	4 (4.8%)
Oropharynx	
Base of tongue	4 (4.8%)
Lateral wall of oropharynx	2 (2.4%)
Posterior wall of oropharynx	2 (2.4%)
Tumor invasion *n* (%)^b^	
Tis	57 (68.7%)
T1	22 (26.5%)
T2	3 (3.6%)
T3	1 (1.2%)
Tumor thickness (mm) mean ± SD	0.83 ± 0.64
Treatment results	*n* = 83
Operation time (min) mean ± SD	89.6 ± 59.6
En bloc resection *n* (%)	83 (100%)
R0 resection *n* (%)	49 (59%)
Adverse events *n* (%)	5 (6.0%)
Post-operative bleeding	2 (2.4%)
Laryngeal edema requiring prolonged intubation	1 (1.2%)
Facial iodine dermatitis	1 (1.2%)
Severe deterioration of swallowing function	1 (1.2%)

^a^Data was missing for 20 patients

^b^Definitions are according to Japanese Clinical Practice Guideline for Head and Neck Cancer

**Table 2:** Bivariable analysis on risk factors associated with positive margins

	R0 rate (%)	*p* value
Patient factors		
Alcohol consumption (g/day)		0.046
≤ 100	50.8	
101–200	64.3	
≥ 201	100.0	
Flusher^a^		0.586
Yes	55.9	
No	65.2	
Active drinker		0.307
Yes	59.7	
No	45.0	
Smoking amount		0.056
< 20	65.5	
21–40	35.3	
≥ 41	41.7	
Smoking years		0.271
< 20	53.6	
21–40	50.0	
≥ 41	71.4	
Active smoker		0.020
Yes	80.0	
No	49.2	
History of esophageal cancer		1.000
Yes	55.9	
No	57.9	
Lesion factors		
Efficacy of laryngeal elevation		0.080
Effective	58.3	
Not effective	0.0	
Clear demarcation with NBI		0.600
Yes	60.0	
No	50.0	
Clear demarcation with Iodine		0.571
Yes	62.8	
No	52.9	
Method used for demarcation		0.025
NBI = Iodine	73.0	
NBI	45.2	
Iodine	42.1	
Location		0.011
Piriform synus	50.0	
Posterior wall of hypopharynx	100.0	
Postcricoid	100.0	
Base of tongue	25.0	
Lateral wall of oropharynx	100.0	
Posterior wall of oropharynx	100.0	
Close vicinity to the glottis		0.397
Yes	33.3	
No	58.0	

^a^Data was missing for 20 patients

## Discussion

In this retrospective single-center analysis, we have demonstrated that pharyngeal ESD is effective and safe, with short-term and long-term results comparable to previous reports [18]. In addition, this is the first report on risk factors other than location which can influence R0 resection rates, and the first to suggest that methods for diagnosis of extent of superficial pharyngeal cancer may significantly influence resection results.

Concerning endoscopic diagnosis of superficial pharyngeal cancer, the use of optical enhancement with NBI has been reported to be effective in detecting superficial pharyngeal cancer and has raised awareness of pharyngeal cancer among GI endoscopists [1, 2]. However the proportion of patients with superficial pharyngeal cancer is still low, with only 5.3% discovered at Tis stage [13], even in Japan where there is an established upper GI endoscopic screening program on a national level [19]. While there are many factors related to the low detection rate, such as the complex structure of the pharynx, low patient compliance, low attention to pharyngeal cancer, and lack of guidelines for screening of pharyngeal cancer, there is also a possibility that optical enhancement alone may not enable visualization of all superficial pharyngeal cancers. The results of this study suggest that although NBI is effective, evaluation of the horizontal extent of pharyngeal cancer may not be completely accurate with only NBI, and endoscopists should be aware of this possibility when performing local resection of superficial pharyngeal cancer.

Iodine staining is another effective method for the diagnosis and evaluation of the horizontal extent of pharyngeal cancer. However, the efficacy of this method is limited by the fact that it cannot be used for screening of non-intubated patients due to the risk of chemical pneumonitis in the event of iodine aspiration, which may occur even in intubated patients when used near the glottis [14]. In addition, iodine staining may not be effective for the accurate evaluation of horizontal extent of lesions with dysplasia or inflammation in the surrounding pharyngeal mucosa [20]. Despite these limitations, at present, otolaryngologists and gastroenterologists must decide on the target area for resection using these two modalities with the objective of obtaining the highest possibility of R0 resection [15]. In cases with disparity between NBI and iodine staining, which were associated with a low R0 resection rate in this study, resection of a wider area may lead to a higher R0 resection rate. However, resection of a wider area also increases the risk of severe adverse events, including irreversible deterioration of swallowing function as shown in this study. Thus, there is a need for more accurate methods of evaluation of superficial pharyngeal cancer. In addition, physicians must be aware of the pros and cons of resection of wider areas, and the necessity of deciding whether to prioritize R0 resection or long-term pharyngeal function on a case-by-case basis when deciding on the target area for resection.

Concerning the technical features of pharyngeal ESD, R0 resection rates for pharyngeal ESD have been consistently reported to be lower than for esophageal ESD, despite using similar methods for the diagnosis of extent of squamous cell carcinoma in the same institutes [21–23]. This is in part due to the technical difficulty of pharyngeal ESD, which is strongly affected by the accessibility of the target lesion, availability of adequate maneuverable space, and mutual interference between the endoscope, laryngoscope and intubation tube. Especially, the complex structure of the pharynx has been reported to be a risk factor for incomplete resection, and different protocols have been suggested for different areas in the pharynx [21]. The results of this study concur with these reports, with 100% R0 resection rates in straight areas of the pharynx, compared to lower rates in areas with complicated structures. Although the efficacy of different protocols for different areas could not be evaluated in this study, Iizuka et al. have demonstrated excellent results with this strategy and this approach seems very promising [21].

However, while achieving R0 resection is an important goal, we have demonstrated that the risk of local recurrence even after non-R0 resection is 8.8%, for a total local recurrence rate of 3.6% after all pharyngeal ESD procedures. Both the R0 resection rates and local recurrence rates in this study are comparable to previous reports, confirming that there is a low recurrence rate even after non-R0 resection, and a wait and see strategy is a feasible option for these cases [6, 12, 21, 24]. However, the risk of local recurrence is not negligible, and continual follow-up after incomplete resection is necessary. Especially for areas which cannot be easily visualized with laryngoscope/endoscope, it is difficult to detect local recurrence even with follow-up at regular intervals, and special care is required after non-R0 resection in these areas.

Concerning the long-term prognosis after pharyngeal ESD, we were able to demonstrate excellent results with a 5-year metastasis rate of 0%, 5-year disease specific survival of 100%, and a 5-year overall survival of 87.0%. However, disease-specific survival and overall survival are obviously strongly the selection bias in choosing cases to be treated by ESD. There are no established guidelines for indications of ESD, and selection of cases to be treated by ESD is largely affected by institutional experience. Although pharyngeal ESD was performed for lesions up to T3 in this study, significant selection bias is expected, and further evidence on the long-term prognosis after ESD is required.

Finally, concerning surveillance after pharyngeal ESD, we found a high 5-year metachronous head and neck cancer rate of 27.1%. These results concur with the well-known “field cancerization” concept in head and neck cancer [25], and careful follow-up even after curative resection with pharyngeal ESD is essential. The results of this study demonstrate that by continuing regular follow-up with both laryngoscopes and upper GI endoscopes after primary treatment, it is possible to maintain a high 5-year overall survival rate despite the high rate of metachronous cancer. Close cooperation between otolaryngologists and gastroenterologists is essential for both treatment and surveillance of head and neck cancer.

There were several limitations to this study. This was a retrospective single center study with a limited number of patients, with selection bias incurred especially during selection of “endoscopically resectable” lesions. In addition, selection of countertraction devices, and assessment of whether iodine staining could be safely performed were decided at the discretion of the operators, and there is a possibility of bias in these factors as well. Further studies are needed in order to standardize indications and strategies for pharyngeal ESD, and to earn a consensus on criteria for curative resection for pharyngeal cancer with ESD.

In conclusion, pharyngeal ESD is effective and safe, and with appropriate postoperative follow-up can enable a high 5-year overall survival rate despite the high rate of metachronous cancer. Further studies are needed to improve and standardize indications and strategies for pharyngeal ESD.
